# Isolation of a New Infectious Pancreatic Necrosis Virus (IPNV) Variant from a Fish Farm in Scotland

**DOI:** 10.3390/v13030385

**Published:** 2021-02-28

**Authors:** Jessica Benkaroun, Katherine Fiona Muir, Rosa Allshire, Cüneyt Tamer, Manfred Weidmann

**Affiliations:** 1 Institute of Aquaculture, University of Stirling, Stirling FK9 4LA, UK; jessica.benkaroun@stir.ac.uk (J.B.); k.f.muir@stir.ac.uk (K.F.M.); rosa.allshire@stir.ac.uk (R.A.); 2 Department of Virology, Faculty of Veterinary Medicine, Ondokuz Mayis University, 55139 Samsun, Turkey; cuneyt_tamer@hotmail.com; 3 Medizinische Hochschule Brandenburg Theodor Fontane, 01968 Senftenberg, Germany

**Keywords:** infectious pancreatic necrosis virus, IPNV, evolution, phylogeny, variant, aquaculture

## Abstract

The aquatic virus, infectious pancreatic necrosis virus (IPNV), is known to infect various farmed fish, in particular salmonids, and is responsible for large economic losses in the aquaculture industry. Common practices to detect the virus include qPCR tests based on specific primers and serum neutralization tests for virus serotyping. Following the potential presence of IPNV viruses in a fish farm in Scotland containing vaccinated and IPNV-resistant fish, the common serotyping of the IPNV isolates was not made possible. This led us to determine the complete genome of the new IPNV isolates in order to investigate the cause of the serotyping discrepancy. Next-generation sequencing using the Illumina technology along with the sequence-independent single primer amplification (SISPA) approach was conducted to fully characterize the new Scottish isolates. With this approach, the full genome of two isolates, V1810–4 and V1810–6, was determined and analyzed. The potential origin of the virus isolates was investigated by phylogenetic analyses along with tridimensional and secondary protein structure analyses. These revealed the emergence of a new variant from one of the main virus serotypes, probably caused by the presence of selective pressure exerted by the vaccinated IPNV-resistant farmed fish.

## 1. Introduction

Infectious pancreatic necrosis virus (IPNV) is an economically relevant pathogen of farmed Atlantic salmon (*Salmo salar Linnaeus* 1758) and rainbow trout (*Oncorhynchus mykiss Walbaum* 1792). The aquatic virus (Birnaviridae) has a bi-segmented double-stranded RNA genome of about six kilobases (kb). Segment A encodes a large polyprotein composed of the major capsid viral protein VP2, the minor capsid protein VP3, and the serine-lysine protease VP4. A second ORF encodes the nonstructural VP5 protein. Segment B encodes the RNA-dependent RNA polymerase (RdRp/VP1) [1,2,3,4,5].

The VP2 protein contains a variable domain in the central region between the amino acid residues 183–335, which is an important antigenic site with two hypervariable regions at residues 239–257 and 271–284 [6]. Amino acid residues at position 217 and 221 have been shown to be linked to virulence types (virulent (T217, A221), persistent (P217, T221), avirulent (T217, T221) and low virulent (P217, A221)) and IPNV is classified into seven genogroups (I-VII) according to 10 known serotypes [6,7,8,9,10,11,12,13]. It should be noted that discrepancies among studies regarding the virulence profile of VP2 have been highlighted in a recent review from Dopazo [14]. It suggests that genetic factors from the host and environmental factors linked to the geographic location could contribute to different degrees of VP2 virulence connected to different different amino acid patterns.

IPNV vaccines include the main immunogenic protein VP2 and other components of the virus [15]. The protection shown in the trials reached more than 80%, but despite the introduction of IPNV vaccines to the farmed stock in 1995, IPNV outbreaks were still observed [16]. IPNV outbreaks in Norwegian aquaculture dropped from 2011 onward when IPN-resistant commercial salmon strains with an identified quantitative trait locus (QTL) became available [17,18]. IPNV-resistant fish seem to be more effective for disease control than vaccines, as a protective immune response in salmon can show large variations depending on their genetic background and the IPNV strains involved [19].

IPNV diagnosis is mostly based on the isolation of the virus from cell culturing followed by a neutralization test to identify the virus serotype, mostly Sp (Spajarup) and Ab (Abild) in Europe, which corresponds to genotype 5 and 2, respectively [20]. In 2018, we obtained tissue samples from the spleen and kidney of Atlantic salmon from an aquaculture farm in Scotland. The histological analyses of the fish tissues showed the hallmark of an IPNV infection, such as liver necrosis, caecal epithelial sloughing and pancreatic acinar necrosis [21]. However, lesions on the pancreas were mild, and another pathology was observed, including gill epithelial separation with degenerative lesions, as well as hydropic degeneration in the liver. Subsequent bacteriology diagnostic analyses revealed no significant findings, but onsite q-RT–PCR testing of 4 samples confirmed high levels of an IPNV (Ct < 20). Onsite overall fish mortality was less than 2%; the outbreak lasted for approximately four weeks. The fish samples were inoculated onto CHSE-214 cells for potential virus isolation and subsequent characterization. The presence of two IPNV isolates was confirmed by RT–PCR from RNA extracts from cell supernatants. After successful virus isolation, the two isolates, V1810–4 and V1810–6 were serotyped using the virus neutralization assay method. Interestingly, the new Scottish isolates did not react with the classical serotype Ab and Sp sera. This led us to hypothesize whether the new isolates could be a new antigenic variant or a virus recombinant. To test both hypotheses, we determined the complete genome of the new IPNV isolates by next-generation sequencing (NGS) along with phylogeny analyses and protein structure observations.

## 2. Materials and Methods

### 2.1. IPNV Isolation by Cell Culture

Tissue from the spleen and kidney were extracted from farmed Atlantic salmon fish. Farmed fish were all QTL-resistant and vaccinated against IPNV. Tissue samples were inoculated on CHSE-214 cells, and culture was performed at 22 °C, 4% CO_2_ in Eagle’s minimum essential medium (EMEM) with 10% fetal bovine serum (FBS) 1× non-essential amino acids and 2 μM l-glutamine. Cells were examined for cytopathic effect (CPE) daily. When a full CPE was visible, the supernatant was collected and stored at −70 °C or used immediately for downstream applications.

### 2.2. IPNV Serotyping by Neutralization Assay

For serological characterization and confirmation of the IPNV isolates, the alpha procedure neutralization test was applied with antisera against the Sp and Ab viral serotypes [22]. The virus was titrated in duplicate with Hank’s balanced salt solution (HBSS) along with an Sp and Ab antisera. After 1 h incubation at 15 °C with a 2% CO_2_ atmosphere, the mixtures were simultaneously inoculated with CHSE-214 cells. The inoculated cells were incubated for a maximum period of 18 days. The neutralization antibody titers were calculated by the method of Spearman–Karber and expressed as the reciprocal of the highest antiserum dilution protecting 50% of the inoculated wells [23,24].

### 2.3. RNA Extraction

Cell supernatant containing the virus was filtered with a 0.2 μm sterile filter prior to RNA extraction with the Agencourt RNAdvance blood kit (Beckman Coulter Life Sciences) according to the manufacture protocol.

### 2.4. IPNV Detection

An in-house q-RT–PCR method was used on the LightCycler 480 RNA master hydrolysis probes kit (Roche, UK) was used with IPNV RNA extracts along with an RNA standard and the following specific primers and probe: forward primer IPNV VP2 FP: 5′-GCCTACCCCCCGTTCCT-3′, reverse primer IPNV_VP2_RP: 5′-CCCGTCACTGTTGTTGAGTTGA-3′, probe IPNV VP2 P2:5′-6FAM-ACTCTCTACGAGGGAAACGCCGAC—BBQ-3′, (TIB Molbio). The assays were run on the LightCycler 480 device at the following cycling conditions: 63 °C for 3 min, 95 °C for 30 s, 45 cycles at 95 °C for 5 s, 60 °C for 15 s, and a final step at 40 °C for 40 s. RT–PCR reactions were performed with two different sets of primers from the cDNA reverse transcribed from the RNA isolates with the high-capacity cDNA reverse transcription Kit (Thermo Fisher Scientific, UK). PCR reactions were performed with MyTaq (Bioline) with the forward primer PrB1:5′-GCCGACATCGTCAACTCCAC-3′ and the reverse primer PrB2:5′-GACAGGATCATCTTGGCATA-3′ (TIB Molbio, Germany) at the following conditions: 34 cycles at 94 °C for 30 s, 58 °C for 1 min, at 72 °C for 1 min, and one cycle at 72 °C for 2 min. With the forward primer: IPN AF-: 5′-ACGAACCCCCAGGACAAG-3′, and the reverse primer IPN AR: 5′-TTGACCCTGGTGATCGGCTT-3′ (TIB Molbio) with the following conditions: 34 cycles at 94 °C for 30 s, 54 °C for 1 min, at 72 °C for 1 min, and one cycle at 72 °C for 2 min.

### 2.5. SISPA Method for the Amplification of IPNV Genomes

*1st strand cDNA synthesis:* first-strand cDNA synthesis from the IPNV RNA was performed using the SunScript reverse transcriptase kit (Expedeon, UK) using 0.5 pmol of SISPA Primer A: 5′-GTT TCC CAC TGG AGG ATA-(N9)-3′ [25]. The reactions were incubated at 65 °C for 60 min and at 95 °C for 10 min to stop the reactions.

*2nd strand cDNA synthesis/Klenow reaction:* The reaction was performed with the DNA polymerase I, large fragment kit (New England Biolabs) according to the manufacturer instructions. The Klenow reactions were incubated for 15 min at 25 °C, at 37 °C for 60 min, and at 75 °C for 20 min to stop the reactions.

*IPNV cDNA enrichment by PCR amplification:* The IPNV cDNA samples were enriched by PCR using MyTaq DNA polymerase (Bioline). All reactions were performed with 0.2 pmol of SISPA_Pirmer_B (10 µM): 5′-GTT TCC CAC TGG AGG ATA-3′. The reactions were incubated at 95 °C for 2 min as an initial denaturation of the cDNA template, followed by 35 cycles of the following steps: 95 °C for 15 s, 50 °C for 15 s, 72 °C for 10 s, and a final extension for 5 min. The PCR products were cleaned with Ampure XP beads (Beckman Coulter Life Sciences, UK).

### 2.6. Library Preparation and Illumina Sequencing

Libraries were prepared with the Illumina Nextera XT DNA library preparation kit (Illumina). 0.5 ng of each IPNV amplicon was used for the tagmentation reaction. The quality and quantification of each library were assessed using the high-sensitivity DNA chip and reagents (Agilent). Libraries were normalized at equimolar concentration and pooled manually. Paired-end sequencing was performed with a read length of 2 × 75 bp using the MiSeq Reagent Kit v3, 150 cycles (Illumina) on the MiSeq sequencer (Illumina, UK).

### 2.7. Data Processing

The quality of raw sequencing reads was checked with the FastQC tool [26]. Illumina adapter sequences were clipped, low-quality reads were filtered and removed with Trimmomatic v.0.39 [27]. Overrepresented sequences were removed with Cutadapt v3.1 [28]. Filtered reads were de novo assembled using SPAdes v.3.15.0 [29]. The resulted contigs were blasted against the nucleotide database from NCBI (National Center for Biotechnology Information) [30].

### 2.8. Phylogenetic Analysis

Highly similar IPNV nucleotide sequences to the new Scottish isolates were retrieved by BLAST on NCBI. All IPNV nucleotide sequences were aligned with MUSCLE implemented in MEGA v.6 [31]. The phylogenetic tree was inferred in MEGA with the neighbor-joining method with 1000 bootstrap replications. Evolutionary distances were computed using the maximum composite likelihood method and are represented by the number of base substitutions per site, scale bar. The evolutionary trees were visualized in MEGA.

## 3. Results

**Figure 1:**
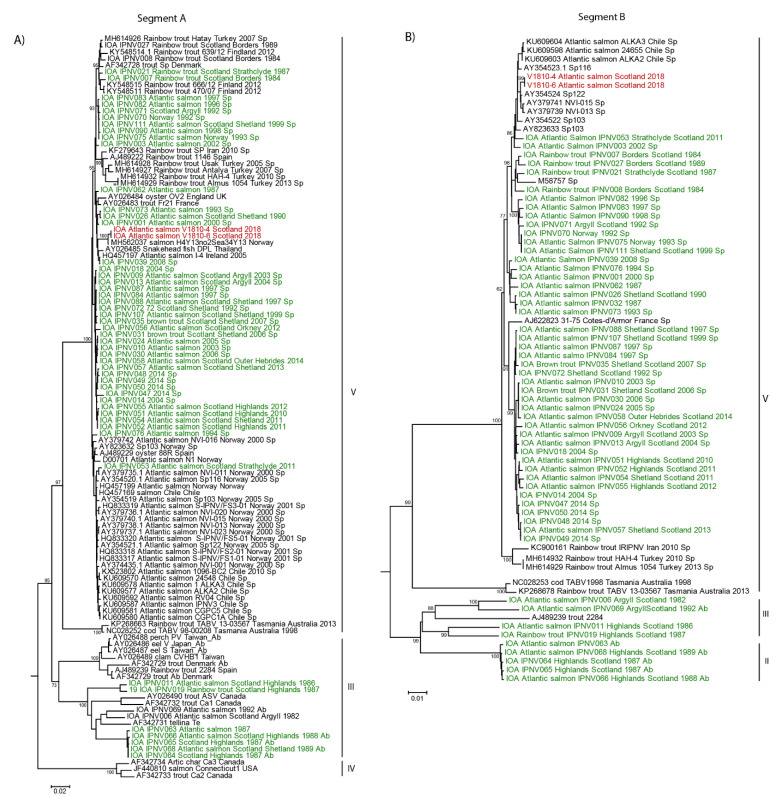
Phylogeny of the new Scottish infectious pancreatic necrosis virus (IPNV) isolates V1810. Nucleotide sequences of the new isolates from Scotland, V1810–4 and V1810–6, were aligned with previous Scottish isolates and similar sequences retrieved by BLAST on NCBI. The alignment was conducted by MUSCLE implemented in MEGA 6. The phylogenetic tree was inferred in MEGA6 with the neighbor-joining method. Bootstrap percentages >70 are indicated. (**A**) Phylogenetic tree of IPNV segment A. (**B**) Phylogenetic tree of IPNV segment B. Sequences on the trees colored in red correspond to the new Scottish isolates V1810–4 and V1810–6; in green are previous Scottish isolates, and in black are sequences retrieved by BLAST on NCBI.

In order to investigate the cause of the serotyping discrepancy of the new Scottish isolates, their genomes were analyzed with additional IPNV sequences deposited on NCBI along with the IPNV Scottish isolates we previously characterized [12]. Phylogenetic analysis of nucleotide sequences revealed that segment A of the new Scottish isolates grouped with an IPNV isolate from Norway isolated between 2004 and 2015, sequence MH562037 with 99.6% nucleotide identities (Figure 1A). The segment B of both Scottish isolates also appeared closely related to sequences determined in previous IPNV outbreaks in Norway, sequences AY354524, AY37974, AY379739, AY354522, and AY823633 (Figure 1B).

Both segments of the new isolates belong to genotype V, which comprises viruses from a variety of geographic locations (Figure 1A,B). This analysis revealed that segment A of these new isolates from Scotland forms a subclade among IPNV sequences of serotype Sp (Figure 1A). This suggests the isolates of this new subclade are emerging variants resistant to Sp-specific antibodies since they were not detected as Sp serotype based on our neutralization test (Supplementary Table S1). This could have happened possibly through mutations accumulating at the antigenic site of the VP2 protein, which is recognized by specific Sp or Ab antibodies.

**Figure 2:**
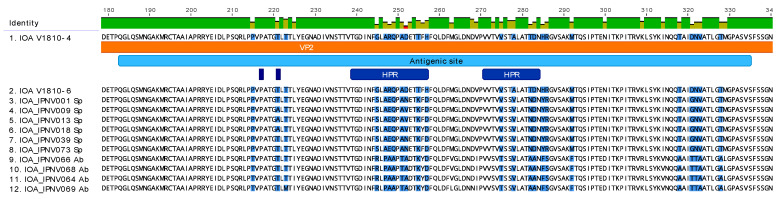
Analysis of the VP2 protein sequences of the new Scottish IPNV isolates at the antigenic site. The new Scottish isolates V1810–4, and V1810–6 amino acid sequences were aligned with Scottish isolates of serotype Sp or Ab with MUSCLE. The sequence was annotated manually in Geneious Prime v.2021.0.3 [32]. Zoom representation of the VP2 protein at the antigenic site. The antigenic site is contained in the VP2 protein (colored in clear blue), HPR regions are colored in dark blue. Amino acid residues variation is highlighted in blue on the sequences.

Analysis of the protein sequences of the new IPNV isolates from Scotland with Sp, or Ab serotype protein sequences showed several amino acid variations appearing at the antigenic site of the VP2 protein in the hypervariable regions (HPR) (Figure 2). Within both HPR of the antigenic site of VP2, the new Scottish isolates contain unique amino acid residues in comparison to the other serotype Sp or Ab sequences (Figure 2, Table 1). When looking at residues at positions 217 and 221 described to be involved in the pathogenicity of the virus (Figure 2), the new variants possess a proline and a threonine, respectively, which are a hallmark of IPNV persistent strains.

**Figure 3:**
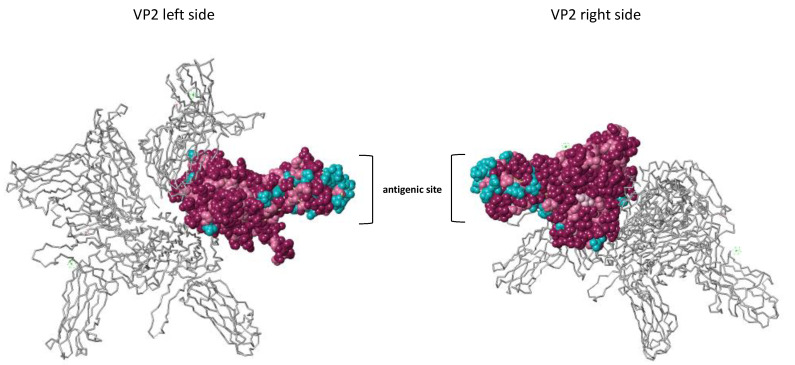
3D structural analysis of the VP2 protein. The conservation of the amino acid residues was estimated using the ConSurf Server [33] and based on the multiple alignments of Sp and Ab serotypes along with the 3IDE_1 PDB 3D structure. The left and right view of the protein is represented. One VP2 monomer is colored on the 3D structure of VP2, which is composed of 4 monomers. Conserved amino acid residues are colored in purple. Pink residues, less conserved than purple residues, are colored in pink. The least conserved residues are colored in blue.

To better highlight the differences between the new Scottish variants and the other isolates from the Sp serotype at their VP2 antigenic site, we conducted an analysis of the three-dimensional structure of the protein VP2 generated from the multiple alignments of Sp and Ab serotype protein sequences (Figure 3). The observation of the 3D structure of the VP2 construction showed that most of the less conserved amino acids are grouped at the antigenic site of the protein, indicating the putative site of recognition of antibodies specific for the Sp and Ab serotypes (amino acid residues colored in blue).

**Figure 4:**
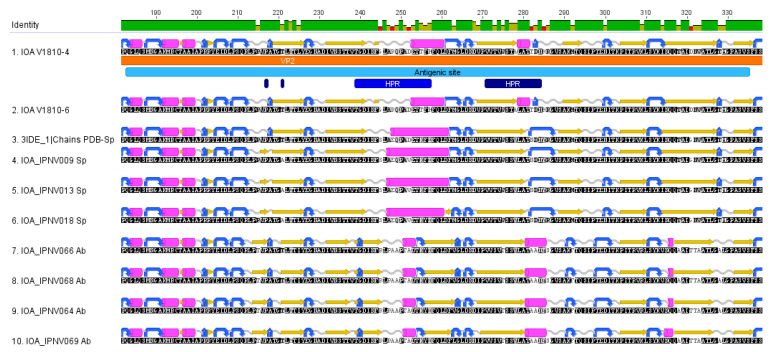
Secondary structures of VP2 proteins. The secondary structures of VP2 proteins among the Sp and Ab isolates and the new V1810–4 and V1810–6 isolates are depicted as follows: Beta sheets are colored in yellow, turns in blue, alpha-helices in pink, and coil structures in clear gray. The secondary structures were predicted with EMBOSS implemented in Geneious Prime. All amino acid sequences are oriented from the amine terminal extremity to the carboxy-terminal extremity.

Exploring the secondary structures of the VP2 proteins of the new Scottish isolates along with VP2 proteins of the Sp and Ab serotypes shows conservation of amino-acid structures within each serotype and among our two new isolates (Figure 4). The Ab serotype has more conserved turn structures (colored in blue) at the N-terminal region of the antigenic site compared to the Sp serotype. A longer alpha helix is seen in the Sp serotype at the central region of the antigenic site and an additional alpha-helix at the end of the antigenic site sequence. Distinct differences can be observed for the sequences of the new isolates compared to the other Sp sequences, which contain an additional small beta-sheet (residue 244), a smaller alpha-helix at position 253–260, an additional alpha helix (position 279–281), and a shorter turn at position 283.

## 4. Discussion

IPN vaccines suppress disease symptoms and improve fish survival, but Julin et al. showed that IPNV isolates extracted from vaccinated fish from salmon farms in Norway induced mortalities in unvaccinated populations [34,35]. These observations indicate that IPN-vaccines are non-sterilizing vaccines that do not totally inhibit IPNV replication [36]. IPNV clearly shows a potential to change its virulence pattern in both directions: due to stress to a high virulent type or due to attenuation to a low virulent type, underlining a risk of emergence of new strains in vaccinated salmon [34,35,37,38].

The IPNV isolates from Scotland were not neutralized by either Ab or Sp serum, and their VP2 protein sequences showed extensive variations. Our analyses indicate that the new IPNV isolates from Scotland seem to have evolved at the antigenic site of the VP2 coding region, more particularly in the two hypervariable regions. Unique HPR secondary structures were evident in the new isolates in comparison to the described Sp and Ab serotype sequences.

It is common practice to vaccinate IPN-QTL salmon to attain extra safety since the resistance to IPNV is not an absolute one. Our analyses suggest that the new IPNV variants evolved to counteract the selection pressure exerted by the vaccinated QTL fish. The mutation on antigenic sites of viruses has also been observed for Measles viruses replicating in vaccinated human populations [39]. A recent report in an industry magazine citing the work of Irene Ørpetveit from the Norwegian Veterinary Institute in Norway appears to corroborate our observations [40]. QTL-resistant fish have been selected for their ability to block the virus entry by preventing the virus interaction with a clathrin cell receptor [18]. Since VP2 is thought to be involved in the cell receptor entry, it would be interesting to investigate whether the new amino acid changes in VP2 have an effect on the virus entry step.

This study provides evidence of the emergence of new IPNV virus variants from a fish farm where vaccinated QTL fish were held. It is well known that stressors can decrease the immune response of fishes [38], and it is quite possible that the combination of gaps in protection and stress-induced immune suppression can contribute to the emergence of new IPNV variants. As yet, the new isolates present as the persistent pathogenicity type, but the emergence of pathogenic types is possible [12]. Constant surveillance of farmed salmon to identify new IPNV types is warranted to be able to update existing vaccines, which have been in use for more than 20 years now and to renew diagnostic tools to identify new virus variants. Challenge experiments to characterize the new isolates and their ability to cause disease in vaccinated QTL-fish would allow studying the impact of these viruses to understand how they circumvent the immune response in more detail.

## Figures and Tables

**Table 1:** Amino acid residues present within the antigenic site of the VP2 proteins of the V1810 isolates compare to the Sp and Ab strains. Unique amino acid residues to the V1810–4 and V1810–6 isolates are highlighted in gray. Specific amino acid residues that seem unique to the serotype Sp or Ab can also be observed (amino acid one-letter code used).

	Amino Acid Position	V1810 Isolates	Sp Strain	Ab Strain
**HPR 239–257**	245	G	S	R
	247	A	A	P
	248	R	E	A
	249	Q	Q	A
	251	A	A	T
	252	D	V/N	A
	253	E	E	D
	255	T	K	K
	257	H	D	D
**HPR 271–284**	275	V	V	T
	278	A	V	V
	282	T	N	A
	283	D	D	A

## Data Availability

Not applicable.

## Supplementary Materials

The following are available online at https://www.mdpi.com/1999-4915/13/3/385/s1, Table S1: Virus titers of the new Scottish isolates V1810–4 and V1810–6.
