# Supplementary material for: Isolation of a New Infectious Pancreatic Necrosis Virus (IPNV) Variant from a Fish Farm in Scotland
Source: Viruses. 2021 Feb 28;13(3):385. doi: 10.3390/v13030385 (PMC7997178; doi:10.3390/v13030385)
Supplement: Supplementary file 1 [file viruses-13-00385-s001.pdf]

Supplementary table S1 : Virus titers of the new Scottish isolates V1810-4 and V1810-6

Sample	Titre of virus only	Titre of Virus + Antisera Sp	Titre of Virus + Antisera Ab	Neutralization Index	Score
V1810-4	$10^{7.0}$TCID$_{50}$ /ml	$10^{6.0}$TCID$_{50}$/ml	-	$10^{1.0}$TCID$_{50}$/ml	Questionable
V1810-4	$10^{7.0}$TCID$_{50}$ /ml	-	$10^{6.5}$TCID$_{50}$/ml	$10^{0.5}$TCID$_{50}$/ml	Non-significant
V1810-6	$10^{6.5}$TCID$_{50}$ /ml	$10^{7.0}$TCID$_{50}$/ml	-	$10^{-0.5}$TCID50/ml	Non-significant
V1810-6	$10^{6.5}$TCID$_{50}$ /ml	-	$10^{6.5}$TCID$_{50}$/ml	$10^{0.0}$TCID$_{50}$/ml	Non-significant

The neutralization antibody titers were calculated by the method of Spearman-Karber and expressed as the reciprocal of the highest antiserum dilution protecting 50% of the inoculated wells. A neutralization index of less than 1 log_{10} is considered non-significant; values between 1.0-1.6 log_{10} are questionable; indices over 1.7 log_{10} are significant.
